# P-669. Characteristics of hospitalized adult patients with *hMPV* infection in the post-covid era

**DOI:** 10.1093/ofid/ofae631.865

**Published:** 2025-01-29

**Authors:** Athina Pyrpasopoulou, Charalampos Zarras, Dimitrios Kouroupis, Chrysi Michailidou, Prodromos Soukiouroglou, Panagiotis Pateinakis, Konstantina Mpani, Maria Terzaki, Dimitrios Molyvas, Eleni Vagdatli

**Affiliations:** Hippokration Hospital Thessaloniki, Thessaloniki, Thessaloniki, Greece; Hippokration Hospital Thessaloniki, Thessaloniki, Thessaloniki, Greece; Hippokration Hospital Thessaloniki, Thessaloniki, Thessaloniki, Greece; Hippokration Hospital Thessaloniki, Thessaloniki, Thessaloniki, Greece; Hippokration Hospital Thessaloniki, Thessaloniki, Thessaloniki, Greece; Hippokration Hospital Thessaloniki, Thessaloniki, Thessaloniki, Greece; Hippokration Hospital Thessaloniki, Thessaloniki, Thessaloniki, Greece; Hippokration Hospital Thessaloniki, Thessaloniki, Thessaloniki, Greece; Hippokration Hospital Thessaloniki, Thessaloniki, Thessaloniki, Greece; Hippokration Hospital Thessaloniki, Thessaloniki, Thessaloniki, Greece

## Abstract

**Background:**

Human Metapneumovirus (*hMPV*) is a recently described respiratory tract virus with significant morbidity among children of younger age. The widespread molecular diagnostic testing has identified *hMPV* as an important pathogen of the upper and lower respiratory tract even in adults, especially the older and immunosuppressed. The aim of this study was to identify patients hospitalized with *hMPV* infection and describe their characteristics.

**Figure:**
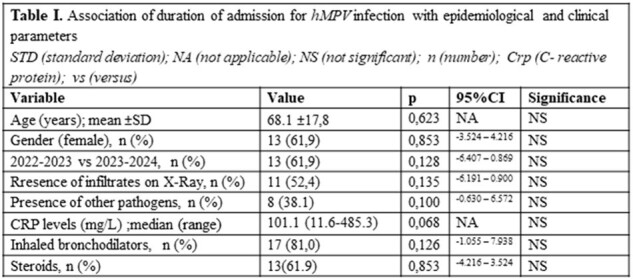

**Methods:**

Rhinopharyngeal samples of admitted patients with respiratory tract infection in the years 2021-22, 2022-23 and 2023-24 from October 1st – March 31st were tested by multiplex PCR analysis (Film array Respiratory panel, Biomerieux, France). Positive patients aged >18 years for hMPV were included in the analysis. Demographic data, clinical and laboratory characteristics were recorded, and correlated with the duration of admission.

**Results:**

In total, 1993 samples were tested (76 in 2021-22, 554 in 2022-23, and 1363 in 2023-24). Among those, 0, 13 and 8 adults were found to be positive respectively, 8 male and 13 female with a mean age of 68,1 years (±17,8). Upon admission, 52,4% of the patients had pulmonary infiltrates on X-Ray. Median CRP was 101,1mg/L (nv < 5mg/L). In 39,1% of the patients another pathogen was isolated from clinical samples (in 4 patients from urine, in 2 from sputum and in 2 from blood cultures). All patients received antibiotics, 61,9% systemic steroids and 81,0% inhaled bronchodilators. One patient aged 94 years died. CRP levels were not associated with the detection of other pathogens (p=0,485). Mean duration of admission (7,3±4 days) was not associated with age. The co-isolation of other pathogens, and CRP levels tended to correlate with longer duration of hospitalization (p=0,100, and p=0,068 respectively), Table I.

**Conclusion:**

*HMPV* is implicated in respiratory tract infections. Clinical presentation is associated with respiratory distress with significantly elevated inflammatory markers, requiring inhaled bronchodilators and systemic steroids. The significant majority of the patients recovers without sequelae.

**Disclosures:**

**Athina Pyrpasopoulou, MD PhD**, Gilead: Honoraria|Pfizer: Honoraria

